# Regional disparities in food security and depression among single-person households in the Republic of Korea

**DOI:** 10.1186/s12889-023-16874-3

**Published:** 2023-10-19

**Authors:** Seong-Ah Kim, Seul Ki Choi

**Affiliations:** 1 Department of Urban Society, The Seoul Institute, Seoul, 06756 Republic of Korea; 2 Graduate School of Urban Public Health, University of Seoul, Seoul, 02504 Republic of Korea

**Keywords:** Single-person households, Food insecurity, Depression, Regional disparities

## Abstract

**Background:**

Single-person households constitute over 40% of all households in the Republic of Korea and are more vulnerable to food insecurity and depression than multi-person households. There is a lack of research on examining whether regional characteristics are associated with the degree of food insecurity and depression among single-person households. This study aimed to examine the regional disparities in food security and depression among single-person households in the Republic of Korea.

**Methods:**

A total of 227,873 adults from the 2019 Korean Community Health Survey was included in the analysis. According to population density and poverty rate, the residence of the participants was classified into four regions: metropolitan areas with high population density were classified into areas with low poverty rates (Region 1) and high poverty rates (Region 2), and provinces with low population density were classified into areas with low poverty rates (Region 3) and high poverty rates (Region 4). Using a single item of household food security, those who had experienced a lack of food due to financial difficulties over the past year were classified as food insecure. Depression was measured using the Patient Health Questionnaire-9. Odds ratios and 95% confidence intervals (CI) for the risk of food insecurity and depression according to regional characteristics were calculated after adjusting for potential confounding variables.

**Results:**

After adjusting for confounding variables, single-person households in regions with high population density, Regions 1 and 2, had 1.16 times (95% CI = 1.04–1.30) and 1.43 times (95% CI = 1.27–1.61) higher odds of food insecurity, respectively, compared to those in Region 4. Single-person households in regions with low poverty rates, Regions 1 and 3, had 1.54 times (95% CI = 1.34–1.77) and 1.21 times (95% CI = 1.01–1.46) higher odds of depression, respectively, than those in Region 4. Among those who lived alone, the middle-aged, having low income, receiving livelihood benefits, or having a low educational attainment had higher odds of experiencing both food insecurity and depression than their counterparts.

**Conclusions:**

As the risk of food insecurity and depression in single-person households differs according to regional characteristics, local governments need to implement policies for single-person households in consideration of these distinct characteristics.

## Background

As family composition has changed from extended families to nuclear families and even smaller families, household composition has changed globally. Over the past several decades, the proportion of single-person households has increased in many developed countries [1]. As with global trends, the proportion of single-person households in the Republic of Korea continues to increase, accounting for 41% of all households [2]. Single-person households may have different resources and needs than multi-person households; thus, they face unique challenges. Previous studies have reported that single-person households are at a heightened risk of food insecurity and social and mental health problems such as loneliness and depression, compared to multi-person households [3–5]. Two recent meta-analyses on food insecurity and depression demonstrated a strong association between food insecurity and depression [6, 7]. One possible underlying mechanism is that food insecurity causes feelings of deprivation and alienation, consequently leading to disrupted social relationships [6, 8]. The interrelationship between food insecurity and depression forms a vicious cycle and increases vulnerability. Thus, it is necessary to examine who are the most vulnerable to both food insecurity and depression and what factors influence the vulnerability.

Food insecurity and depression that single-person households suffer from are closely related to individual-level characteristics, such as age, education level, occupation, and economic status [9–15]. Regional and community characteristics, such as urban or rural classification, proportion of regional welfare budget, and unemployment rate, are also associated with residents’ food insecurity and depression [16, 17]. Despite the potential impact of individual- and regional-level characteristics on food insecurity and depression, there is a lack of studies examining their prevalence according to regional characteristics, especially among single-person households. In addition, the characteristics of single-person households vary by region, but policies and programs for them are similar across regions. For example, in the Republic of Korea, young single-person households mostly live in the capital city, Seoul, or other metropolitan areas where universities and industrial infrastructure are concentrated. In contrast, the proportion of elderly single-person households is higher in non-metropolitan areas than in other regions (Statistics Korea, 2021). Understanding regional-level differences in food insecurity and depression among single-person households would help to develop regional strategies to address these issues. However, there is a lack of studies examining food insecurity and depression together and most studies on single-person households have focused only on older adults. This study aimed to examine regional disparities in food security and depression among single-person households in the Republic of Korea, where the proportion of single-person households is increasing rapidly [2].

## Methods

### Study population

We used the data from the 2019 Korean Community Health Survey (KCHS). The KCHS is a large-scale, community-based, cross-sectional survey conducted by the Korea Disease Control and Prevention Agency. The KCHS data, which include information about sociodemographics, health behaviors, and health status, have been used to improve community health promotion and disease prevention policies and programs. Detailed information regarding the survey is reported elsewhere [18]. In 2019, 229,099 adults aged ≥ 19 years residing in 17 cities and provinces completed the survey. After excluding those with incomplete responses regarding food security (n = 247) and depression (n = 979), 227,873 participants were included in the analysis.

### Regional characteristics and household composition

Local administrative districts in the Republic of Korea, comprising one capital city, seven metropolitan cities (referred to as ‘si’ in Korean), and nine provinces (referred to as ‘do’ in Korean) were classified by their regional characteristics – population density and poverty rate. The classification process involved dividing the administrative districts into regions with high population density (≥ 1,000 persons/km^2^) and regions with low population density (< 1,000 persons/km^2^). Additionally, the regional poverty rate was considered, measured by the proportion of recipients of livelihood benefits from the Basic Livelihood Security Program (BLSP), a social security program for low-income households. BLSP provides tailored benefits spanning livelihood, health, housing, and education categories to eligible households meeting predetermined income and asset criteria. The eligibility criteria for livelihood benefits are the strictest compared to other benefit criteria. Consequently, the percentage of individuals receiving livelihood benefits within a given region could reflect the prevalence of extreme poverty at the regional level. The national average proportion of livelihood benefit recipients was 3.17%, serving as a benchmark for categorization. Regions with a higher proportion of livelihood benefit recipients than the national average were deemed as having high poverty rates, while regions with a lower proportion were classified as having low poverty rates. Subsequently, the local administrative districts were categorized into four regions as follows: Region 1, where population density is high and the poverty rate is low, includes the capital city of Seoul, Gyeonggi-do, which surrounds the capital city, and Ulsan-si, where large industrial parks are located. Region 2, where both population density and poverty rate are high, included Busan-si, Daegu-si, Incheon-si, Gwangju-si, and Daejeon-si, all of which are metropolitan cities. Region 3, where both the population density and poverty rate are low, includes Sejong-si, Chungcheongnam-do, and Jeju-do. Region 4, where the population density is low and poverty rate is high, includes Gangwon-do, Chungcheongbuk-do, Jeollabuk-do, Jeollanam-do, Gyeongsangbuk-do, and Gyeongsangnam-do. No metropolitan cities were included in Region 4.

Household composition was classified into single-person and multi-person households based on the number of household members. A household with one person was classified as a single-person household and a household with two or more members was classified as a multi-person household.

### Food security

To assess household food security status, a representative of each household was asked to choose one of the four responses to the following question: “Which of the following best describes your household’s situation over the past year?”. Those who affirmed, “My whole family could have a sufficient amount and various kinds of food” were classified into the “secure” group. Those who selected “My whole family could eat a sufficient amount of food but could not eat a variety of foods” were classified into “mildly insecure.” Those who affirmed, “My family sometimes lacked food due to financial difficulties” or “My family often lacked food due to financial difficulties” were classified into “moderately/severely insecure” [19, 20]. We considered those who were food secure or mildly insecure as food secure and those who were moderately or severely insecure as food insecure.

### Depression

The Patient Health Questionnaire-9 (PHQ-9), a nine-item measure of depression based on the Diagnostic and Statistical Manual criteria for major depressive disorder, was used to screen for depression status [21]. Each item was scored from 0 to 3 according to the frequency of depressive symptoms experienced during the last two weeks (0 = “not at all” to 3 = “nearly every day”). The total score was calculated as the sum of the nine scores (ranging from 0 to 27). Those with a total score of ≥ 10 were considered to have depression. In the present study, Cronbach’s alpha of the PHQ-9 was 0.80, confirming a good level of internal consistency [22].

### Confounding variables

As confounding variables, the sociodemographic status of survey participants such as sex, age, household income, receipt of livelihood benefits (from the BLSP), education level, and occupation were included. Age was classified into three groups: 19–39 years, 40–64 years, and ≥ 65 years. Household income included all kinds of earning such as earnings from employment, public assistance and benefits, retirement and pension income, interests and dividends, private transfer, etc. Household income was classified into quartiles based on equivalized household income. Equivalized household income was calculated by dividing the average monthly household income by the square root of the number of household members. We categorized individuals according to their experience of receiving livelihood benefits from BLSP into three categories: current recipients, former recipients, and never received. Educational status was classified into three categories: middle school or lower, high school, and college graduate or higher. Occupation was classified into four groups: white-collar, blue-collar, housewives or students, and unemployed. Given that there was no question in KCHS to determine retirement status of respondents, retired people are included in the unemployed category.

### Statistical analysis

Because the KCHS is based on the complex sampling design, appropriate use of sampling weighs and statistical procedures was applied in all analyses. We used sampling weights provided by KCHS, which were constructed considering the weighting factors of households and individuals, taking into account sampling rates based on household and individual characteristics, as well as response rates. Furthermore, the weights were adjusted based on the sex and age distribution of the registered population structure in each surveyed area. Detailed information regarding the sampling weights can be found elsewhere [23].

The general characteristics of participants according to regional characteristics and household composition were compared using weighted percentages, which provided estimates of the population percentage for each category, using PROC SURVEYFREQ. To assess the risk of food insecurity, depression, and the co-occurrence of food insecurity and depression among single-person households according to the regional characteristics, odds ratios (ORs) and 95% confidence intervals (CIs) were calculated using binary multivariable logistic regression analysis (PROC SURVEYLOGISTIC) after adjusting for potential confounding variables. Before conducting regression analysis, multicollinearity was assessed for all confounding variables by variance inflation factors (VIFs). None of the variables showed a correlation coefficient greater than 0.8 with each other or a VIF of 2.5 or higher, indicating absence of multicollinearity. All statistical analyses were performed using Statistical Analysis System (SAS) software (version 9.4; SAS Institute, Cary, NC, USA). Statistical significance was set at p < 0.05.

## Results

Table 1 displays the general characteristics of the study population according to the regional characteristics and household composition. The proportion of single-person households in Region 1 (11.1%) and Region 2 (11.6%), which mostly consisted of metropolitan areas, was less than those in Region 3 (14.4%) and Region 4 (14.6%). Participants living in Region 1 were financially better off, well-educated, and more likely to be white-collar workers than those living in other regions. In contrast, participants living in Region 4 were more likely to have lesser household income and education, and less likely to be white-collar workers. Single-person households in Region 1 were relatively young, whereas those in Region 4 were relatively old.

**Table 1:** General characteristics of study population according to the regional characteristics and household composition

	Total population		Region 1 (High population density and low poverty rate)		Region 2 (High population density and high poverty rate)		Region 3 (Low population density and low poverty rate)		Region 4 (Low population density and high poverty rate)	
n (weighted %)	Single-person households	Multi-person households	Single-person households	Multi-person households	Single-person households	Multi-person households	Single-person households	Multi-person households	Single-person households	Multi-person households
Total	36,224 (12.3)	191,649 (87.7)	8349 (11.1)	60,611 (88.9)	5683 (11.6)	34,137 (88.4)	3238 (14.4)	16,077 (85.6)	18,954 (14.6)	80,824 (85.4)
Sex										
Male	12,576 (46.0)	89,518 (50.1)	3349 (47.1)	27,854 (49.7)	2098 (44.9)	15,475 (49.8)	1179 (49.6)	7599 (50.7)	5950 (44.4)	38,590 (50.9)
Female	23,648 (54.0)	102,131 (49.9)	5000 (52.9)	32,757 (50.3)	3585 (55.1)	18,662 (50.2)	2059 (50.4)	8478 (49.3)	13,004 (55.6)	42,234 (49.1)
Age group										
19–39	5031 (28.7)	44,912 (34.5)	1998 (33.9)	18,289 (36.6)	998 (26.2)	9274 (34.8)	390 (29.0)	3252 (33.4)	1645 (23.0)	14,097 (30.4)
40–64	11,838 (38.1)	92,281 (48.5)	3232 (38.8)	29,800 (48.0)	2057 (39.3)	16,535 (48.9)	1100 (39.0)	7575 (48.2)	5449 (36.1)	38,371 (49.3)
65+	19,355 (33.2)	54,456 (17.0)	3119 (27.2)	12,522 (15.4)	2628 (34.5)	8328 (16.3)	1748 (32.0)	5250 (18.4)	11,860 (40.9)	28,356 (20.3)
Household income										
Q1	20,348 (44.6)	37,379 (13.2)	2990 (34.3)	6138 (9.5)	2928 (48.3)	5606 (14.0)	1801 (46.3)	3700 (17.5)	12,629 (56.0)	21,935 (18.8)
Q2	4306 (13.3)	51,158 (24.4)	1142 (13.9)	13,591 (21.6)	758 (13.8)	9280 (26.0)	379 (11.8)	4512 (26.1)	2027 (12.5)	23,775 (28.0)
Q3	5335 (19.3)	49,251 (28.4)	1769 (22.3)	17,230 (28.6)	1002 (19.6)	9528 (29.6)	439 (17.2)	3974 (27.6)	2125 (15.5)	18,519 (26.9)
Q4	5923 (22.7)	52,624 (34.1)	2328 (29.5)	23,181 (40.3)	942 (18.4)	9516 (30.4)	564 (24.8)	3658 (28.8)	2089 (16.0)	16,269 (26.2)
Livelihood benefits received										
Current	3177 (9.1)	4286 (2.0)	691 (8.1)	1036 (1.6)	725 (12.5)	969 (2.5)	264 (6.9)	359 (2.1)	1497 (8.5)	1922 (2.1)
Former	483 (0.9)	991 (0.4)	55 (0.6)	245 (0.4)	46 (0.9)	173 (0.5)	45 (0.9)	58 (0.2)	337 (1.3)	515 (0.5)
Never	32,543 (90.0)	186,352 (97.6)	7602 (91.3)	59,320 (98.0)	4910 (86.6)	32,994 (96.9)	2928 (92.2)	15,658 (97.6)	17,103 (90.1)	78,380 (97.4)
Educational status										
Middle school or lower	20,656 (35.9)	61,967 (18.7)	3008 (26.6)	12,206 (14.9)	2755 (38.1)	8825 (18.3)	1904 (38.0)	6153 (22.5)	12,989 (46.7)	34,783 (25.4)
High school	8367 (31.7)	66,802 (37.9)	2549 (32.8)	22,995 (37.8)	1614 (32.7)	12,244 (37.3)	761 (33.5)	5352 (37.8)	3443 (28.9)	26,211 (38.5)
College graduate or higher	7146 (32.4)	62,720 (43.4)	2768 (40.4)	25,347 (47.2)	1307 (29.1)	13,055 (44.3)	564 (28.3)	4549 (39.5)	2507 (24.3)	19,769 (36.2)
Occupation										
White-collar	4604 (21.8)	38,188 (27.1)	1889 (28.4)	16,836 (31.5)	828 (18.8)	7503 (26.0)	338 (16.3)	2558 (22.0)	1549 (15.9)	11,291 (20.8)
Blue-collar	14,186 (36.3)	84,514 (37.3)	2657 (32.3)	20,476 (32.6)	1948 (35.0)	12,796 (36.5)	1370 (45.8)	8267 (46.0)	8211 (40.8)	42,975 (44.9)
Housewife, student	8769 (22.5)	41,574 (23.1)	2015 (21.5)	14,818 (23.7)	1616 (24.8)	8703 (24.3)	786 (20.5)	3069 (20.9)	4352 (22.5)	14,984 (21.3)
Unemployed	8599 (19.4)	27,134 (12.5)	1755 (17.8)	8338 (12.2)	1286 (21.3)	5119 (13.2)	733 (17.5)	2147 (11.1)	4825 (20.8)	11,530 (13.0)

Number of missing values was 1549, 215, and 305 for household income, educational status, and occupation, respectively. Weighted percentage (estimates of the population percentage for each category) was calculated using PROC SURVEYFREQ procedures; Equivalized income was obtained by dividing the average monthly household income by the square root of the number of household members.

The proportion of single-person households with moderate/severe food insecurity was highest in Region 2 (13.5%), followed by Region 4 (10.7%), Region 1 (8.1%), and Region 3 (6.6%). The prevalence of depression in single-person households was highest in Region 1 (6.5%), followed by Region 3 (5.5%), Region 4 (5.3%), and Region 2 (5.1%). When food insecurity and depression were considered together, the proportion of single-person households with both was highest in Region 2 (2.3%), followed by Region 3 (1.8%), Region 1 (1.6%), and Region 4 (1.4%). Food security status and prevalence of depression among multi-person households showed the same trend as among single-person households according to region (Table 2).

**Table 2:** Food security and depression prevalence of study population according to regional characteristics and household composition

n (weighted %)	Total population	Region 1 (High population density and low poverty rate)	Region 2 (High population density and high poverty rate)	Region 3 (Low population density and low poverty rate)	Region 4 (Low population density and high poverty rate)
Food security					
Single-person households					
Secure	16,323 (52.4)	4410 (56.6)	2470 (48.5)	1509 (52.4)	7934 (49.1)
Mildly insecure	15,641 (37.7)	3126 (35.3)	2308 (38.0)	1444 (40.9)	8763 (40.1)
Moderately/severely insecure	4260 (9.9)	813 (8.1)	905 (13.5)	285 (6.6)	2257 (10.7)
Multi-person households					
Secure	127,132 (69.8)	42,243 (71.2)	22,044 (68.2)	10,597 (67.3)	52,248 (69.1)
Mildly insecure	58,045 (27.5)	16,733 (26.4)	10,570 (28.3)	5092 (30.7)	25,650 (27.9)
Moderately/severely insecure	6472 (2.8)	1635 (2.4)	1523 (3.5)	388 (2.0)	2926 (2.9)
Depression					
Single-person households	2124 (5.8)	560 (6.5)	319 (5.1)	206 (5.5)	1039 (5.3)
Multi-person households	5335 (2.9)	1796 (3.0)	954 (2.7)	472 (3.0)	2113 (2.8)
Food insecurity & depression					
Single-person households	658 (1.7)	153 (1.6)	148 (2.3)	57 (1.8)	300 (1.4)
Multi-person households	675 (0.3)	192 (0.3)	174 (0.4)	44 (0.2)	265 (0.3)

After adjusting for confounding variables, the odds of food insecurity were 1.16 times (95% CI = 1.04–1.30) and 1.43 times (95% CI = 1.27–1.61) significantly higher for single-person households in Regions 1 and 2, respectively, significantly lower for those in Region 3 (OR = 0.68, 95% CI = 0.57–0.81), compared to those in Region 4. The odds of depression were significantly higher for single-person households in Region 1 (OR = 1.54, 95% CI = 1.34–1.77) and Region 3 (OR = 1.21, 95% CI = 1.01–1.46), respectively, than for those in Region 4. Single-person households in Region 1 had the highest odds of having both food insecurity and depression (OR = 1.64, 95% CI = 1.27–2.12), followed by Region 3 (OR = 1.58, 95% CI = 1.13–2.22), and Region 2 (OR = 1.52, 95% CI = 1.18–1.97) compared to their counterparts living in Region 4. In terms of effect size, low household income was the best predictor of being food insecure among single-person households. Compared to single-person households with the highest household income (Q4), those with the lowest household income had 24 times higher risk of being food insecure (OR = 24.08, 95% CI = 16.43–35.29). Currently or formerly receiving livelihood benefits and lower educational attainment were also significantly associated with food insecurity. The OR for food insecurity was higher among males (OR = 1.42, 95% CI = 1.27–1.59) compared to females. Young adults (19–39 years old) were less likely (OR = 0.74, 95% CI = 0.58–0.94) and middle-aged adults (40–64 years old) were more likely to be food insecure (OR = 1.57, 95% CI = 1.41–1.76) compared to those aged 65 years or older. People living in region 1 (OR = 1.16, 95% CI = 1.04–1.30) and region 2 (OR = 1.43, 95% CI = 1.27–1.61) had higher odds of being food insecure, but those living in region 3 had lower odds of being food insecure (OR = 0.68, 95% CI = 0.57–0.81) than those living in Region 4. The different occupation types had different food security statuses. People who were blue-collar workers (OR = 1.61, 95% CI = 1.17–2.22), housewives or students (OR = 1.97, 95% CI = 1.43–2.73), and unemployed (OR = 2.34, 95% CI = 1.70–3.22) had significantly higher odds of being food insecure than those working as white-collar workers.

The results for depression were similar to those for food insecurity in terms of household income, receipt of livelihood benefits, and educational status. Those with lower household income and educational status, or who currently or formerly received livelihood benefits had significantly higher odds of being depressed than their counterparts. In particular, experience of receipt of livelihood benefits was the best predictor of depression among single-person households (OR = 2.54, 95% CI = 2.17–2.95). While males were more likely to be food insecure, they were less likely to be depressed than females (OR = 0.63, 95% CI = 0.55–0.73). Compared to older adults aged 65 + years, young adults (19–39 years old; OR = 1.71, 95% CI = 1.37–2.12) and middle-aged adults (40–64 years old; OR = 1.32, 95% CI = 1.13–1.54) had higher odds of being depressed. The odds of being depressed differed by residence area and occupation type. Regions 1 and 3, where poverty rates are low had higher odds of depression than Region 4 (Region 1: OR = 1.54, 95% CI = 1.34–1.77; Region 3: OR = 1.21, 95% CI = 1.01–1.46). Those unemployed were more likely (OR = 1.67, 95% CI = 1.29–2.18) to be depressed than white-collar workers.

Experiencing both food insecurity and depression was associated with age, region, household income, receipt of livelihood benefits, and educational status (Table 3). Among those, household income was the most powerful predictor of having both food insecurity and depression among single-person households. Middle-aged adults had 1.85 higher odds (95% CI = 1.45–2.36) of experiencing both food insecurity and depression than older adults aged 65 + years. Compared with people living in Region 4, those living in other regions were significantly more likely to experience both food insecurity and depression; people living in Region 1 had the highest odds (OR = 1.64, 95% CI = 1.27–2.12), followed by those living in Region 3 (OR = 1.58, 95% CI = 1.13–2.22), and Region 2 (OR = 1.52, 95% CI = 1.18–1.97). People with lower household incomes, those who ever received livelihood benefits, and with less education were more likely to be both food insecure and depressed.

**Table 3:** Multivariable adjusted odds ratios and 95% confidence intervals of food insecurity and depression among single-person households

	Food insecurity	Depression	Food insecurity and depression
Sex = male	1.42 (1.27–1.59)	0.63 (0.55–0.73)	1.12 (0.88–1.43)
Sex = female	1.00 (reference)	1.00 (reference)	1.00 (reference)
Age group = 19–39	0.74 (0.58–0.94)	1.71 (1.37–2.12)	0.63 (0.34–1.15)
Age group = 40–64	1.57 (1.41–1.76)	1.32 (1.13–1.54)	1.85 (1.45–2.36)
Age group = 65+	1.00 (reference)	1.00 (reference)	1.00 (reference)
Region = 1 (High population density and low poverty rate)	1.16 (1.04–1.30)	1.54 (1.34–1.77)	1.64 (1.27–2.12)
Region = 2 (High population density and high poverty rate)	1.43 (1.27–1.61)	0.95 (0.81–1.10)	1.52 (1.18–1.97)
Region = 3 (Low population density and low poverty rate)	0.68 (0.57–0.81)	1.21 (1.01–1.46)	1.58 (1.13–2.22)
Region = 4 (Low population density and high poverty rate)	1.00 (reference)	1.00 (reference)	1.00 (reference)
Household income = Q1	24.08 (16.43–35.29)	2.49 (1.94–3.19)	38.33 (7.52-195.37)
Household income = Q2	6.42 (4.28–9.62)	1.71 (1.31–2.23)	9.23 (1.64–51.93)
Household income = Q3	2.88 (1.92–4.34)	1.32 (1.03–1.70)	3.86 (0.74–20.13)
Household income = Q4	1.00 (reference)	1.00 (reference)	1.00 (reference)
Livelihood benefits received = Current recipients	3.61 (3.21–4.07)	2.54 (2.19–2.95)	3.78 (3.02–4.73)
Livelihood benefits received = Former recipients	2.66 (1.93–3.65)	2.15 (1.42–3.25)	3.89 (1.98–7.65)
Livelihood benefits received = Never received	1.00 (reference)	1.00 (reference)	1.00 (reference)
Education = Middle school or lower	1.90 (1.55–2.32)	1.50 (1.18–1.90)	1.71 (1.06–2.75)
Education = High school	1.35 (1.10–1.66)	1.33 (1.08–1.63)	1.43 (0.88–2.33)
Education = College graduate or higher	1.00 (reference)	1.00 (reference)	1.00 (reference)
Occupation = Blue-collar	1.61 (1.17–2.22)	0.81 (0.63–1.03)	0.98 (0.38–2.50)
Occupation = Housewives or students	1.97 (1.43–2.73)	0.93 (0.72–1.20)	1.44 (0.60–3.47)
Occupation = Unemployed	2.34 (1.70–3.22)	1.67 (1.29–2.18)	1.90 (0.76–4.71)
Occupation = White-collar	1.00 (reference)	1.00 (reference)	1.00 (reference)

## Discussion

We examined the prevalence of food insecurity and depression in single-person households according to regional characteristics. The prevalence of food insecurity, depression, and having both food insecurity and depression was higher among single-person households than among multi-person households in all regions, but the prevalence differed according to regional characteristics. Regions with high poverty rates had a higher proportion of food-insecure single-person households than other regions. However, when individual characteristics were adjusted for, the regional poverty rate was not associated with food insecurity among single-person households. Instead, those in regions with high population density were more likely to be food insecure than those in regions with low population density. Among those who lived alone, middle-aged, had low income, ever received livelihood benefits, or had low educational attainment were at risk of experiencing both food insecurity and depression.

Poverty is a well-known predictor of food insecurity [11, 12, 20]. This study showed that the proportion of food-insecure single-person households was higher in regions with high poverty rates. However, when individual characteristics were adjusted, the likelihood of being food insecure among single-person households was associated with regional population density and not with the poverty rate. We speculate that regions with high population densities may affect food insecurity in two ways. First, the age group of residents living alone in high population density regions may be associated with food insecurity. Our study showed that working-aged (40–64 years) group and high population density regions were independent predictors of food insecurity among single-person households. High population density regions are usually urban, especially the centers of business activities. Thus, working-aged people and jobseekers tend to live there or nearby to access job opportunities. In the Republic of Korea, most public and private social security programs, including food assistance programs, are for low-income older adults, the handicapped, and persons with children. Although working-aged persons could benefit from the BLSP, they need to be in extreme poverty to meet the eligibility criteria. Thus, some working-aged people who are in need may not be able to receive assistance. Second, high population density can lead to resource problems. Due to the high number of residents in a limited area, the cost of living, such as housing and food, is usually higher than in other areas. Even though people could receive benefits from the BLSP, the amount of money they receive is the same, regardless of where they live. This means that people living in regions with higher living costs, such as high population density areas, face higher burdens on cost of living than those living in other regions. The most recent survey regarding housing in Korea reported that people living in a capital city or nearby (Region 1 in this study) are required to pay 10 times their annual income to buy a house, while those living in rural areas (Regions 3 and 4 in this study) pay 4 times their annual income. The cost of renting a house also differs by region; individuals living in a capital city or nearby area pay 17.8% of their monthly income for rent, while those living in rural areas pay 12.6% of their monthly income [24]. To supplement the higher housing costs in urban areas, a part of the BLSP provides housing benefits according to the region, but it is insufficient. A review article reported the potential protective effect of rural living on food insecurity. Seven of the eleven studies reviewed showed that rural living was inversely associated with food insecurity [25]. The protective effect of living in rural or low population density regions may be due, in part, to a relatively low cost of living. Thus, individuals living in urban/high population density areas have less money for food because of the high cost of living than those living in rural/low population density areas. The protective effect of living in rural areas could be the same for both single-person and multi-person households. Our analyses revealed the same effects (data not shown). Third, living alone may lead to high expenses. We speculate that the burden of cost of living would be higher among single-person households than among multi-person households. Multi-person households can benefit from economies of scale. For example, the same place requires the same housing and maintenance costs, regardless of the number of people living there. Thus, individuals living alone may have higher expenses per person than those living with others, especially those living with multiple individuals earning income. This result implies that regional strategies to address food insecurity among single-person households need to consider not only poverty, but also other regional characteristics, such as population density.

The prevalence of depression among single-person households is higher in regions with low poverty rates. Given the relationship between poverty and mental health [26], we expected that depression prevalence among single-person households would be higher in regions with high poverty rates. However, regional poverty was inversely associated with depression in single-person households. Previous studies of the association between mental health and regional poverty have reported inconsistent results. Several studies have reported that living in low-income neighborhoods is associated with depressive symptoms [16, 27, 28]. Other studies have demonstrated an association between relative deprivation or income inequality and mental health [29, 30]. This mechanism can be explained by social comparisons. Individuals living in high-income regions may perceive relative deprivation, which can cause negative psychological responses, including depression [29, 31, 32]. Most previous studies on relative deprivation and mental health did not account for the number of household members. Additional work is needed to investigate whether single-person households are more vulnerable to depression due to relative deprivation than multi-person households.

This study highlighted that single-person households are more vulnerable to experiencing both food insecurity and depression than multi-person households. Among those, middle-aged, low-income, current or former livelihood benefits recipients, or lower-educated were especially high at risk of experiencing food insecurity and depression. These predictors of having both food insecurity and depression were similar to the predictors of independent food insecurity and depression, except for sex, young age (19–39 years old), and occupation. This implies that food-insecure individuals, especially those living alone, need food assistance as well as mental health support, and vice versa. Organizations providing food assistance and mental health services need to collaborate in screening, providing appropriate services, and transferring to other services for those at risk of complex issues such as food insecurity and depression.

In this study, working-aged individuals who lived alone experienced food insecurity and depression compared to older adults. Older adults are known to be at risk of food insecurity or depression; however, when we analyzed only single-person households, the results were inconsistent with those of previous studies. This may be due in part to the lack of safety nets for working-aged people. As mentioned earlier, social security benefits for working-aged persons in the Republic of Korea are less compared to well-known disadvantaged groups, such as low-income older adults, children, and their mothers. Thus, working-aged people who experience deprivation have insufficient resources to overcome their difficulties. This can lead to multiple deprivation, including food insecurity, and generate other kinds of difficulties such as mental health issues. Strategies to address food insecurity and depression among working-aged single-person households that have received less attention are needed.

This study has several limitations. First, given that regional characteristics were limited to population density and poverty rate, we might have missed other area-level characteristics that may be associated with food insecurity. Previous studies have suggested that local social environments are associated with food insecurity [33, 34]; however, we did not consider the social characteristics of each region. We also did not consider charitable food assistance or community initiatives to address food insecurity and the available mental health services in each region. While there are different types of unemployment including voluntary unemployment, involuntarily job loss, and retirement, KHCS did not distinguish between these statuses. Further research need to explore the diverse perspectives of unemployment status and its association with food insecurity and depression.

## Conclusions

To the best of our knowledge, this is one of the first studies to examine the factors associated with the dual burden of food insecurity and depression, considering the characteristics of where individuals live. Given that both food insecurity and depression are important public health issues and that single-person households are increasing, efforts to address food insecurity and depression in single-person households should be continued.

Food insecurity is strongly associated with household income [11, 12, 20]; thus, most policies addressing food insecurity tend to focus on high-poverty regions. This study showed that the crude proportion of single-person households experiencing food insecurity was higher in high-poverty regions, but high population density was also associated with the regional level of food insecurity when residents’ characteristics were adjusted. This means that approaches to address food insecurity need to consider not only high-poverty regions, where a higher number of food insecure people live, but also tailored approaches to meet regional characteristics. This is the same for approaches to address the mental health issues of residents. However, there are no universal one-size-fits-all interventions across different regions, within the same country. Local governments need to consider regional characteristics when preparing policies for single-person households.

## Data Availability

The datasets generated and analyzed during the current study are available from the corresponding author upon reasonable request. The raw data of the 2019 Korea Community Health Survey used for this study can be obtained from the following: https://chs.kdca.go.kr/chs/rawDta/rawDtaProvdMain.do.

## Abbreviations

KCHS: Korean Community Health Survey
PHQ-9: The Patient Health Questionnaire-9
OR: Odds Ratio
CI: Confidence Interval
SAS: Statistical Analysis System
